# Integrated analysis of single-cell and bulk RNA sequencing data reveals immune-related lncRNA-mRNA prognostic signature in triple-negative breast cancer

**DOI:** 10.1016/j.gendis.2023.04.006

**Published:** 2023-05-10

**Authors:** Hongying Zhao, Lei Yu, Lixia Wang, Xiangzhe Yin, Kailai Liu, Wangyang Liu, Shihua Lin, Li Wang

**Affiliations:** a College of Bioinformatics Science and Technology, Harbin Medical University, Harbin, Heilongjiang 150081, China; b College of Biomedical Information and Engineering, Hainan Medical University, Haikou, Hainan 571199, China

Triple-negative breast cancer (TNBC) is an aggressive subtype characterized by extensive tumor heterogeneity, with limited treatment options and a poor prognosis.^1^ We used cell type annotation and trajectory analysis to identify two distinct differentiation states of epithelial cells, while tumor cells predominantly resided on one of these branches. Inferring the malignant state of epithelial cells based on copy number variation, 280 tumor cells were eventually identified from epithelial cells. Differential genes between tumor cells and non-tumor cells were mainly enriched in immune-related functions and pathways. To explore the immune roles of differential genes, we finally identified 106 key lncRNAs and mRNAs associated with immune processes by integrating single-cell and bulk RNA-seq data from TNBC. Moreover, based on the LASSO-COX prognostic model, we systematically identified five lncRNAs and six mRNAs that were significantly associated with TNBC survival (Fig. S1). The immune-related 11 lncRNA-mRNA signature can be used as a prognostic signature to independently divide the overall survival time of patients. Our immune-related prognostic signature has prognostic significance for 15 cancers and exhibited excellent predictive performance compared to the 20 prognostic models, indicating that it may have clinical value in improving the overall survival of patients.

TNBC scRNA-seq data contained a total of 13,280 genes, 8186 differentially expressed genes were identified from 14 clusters, and the 14 clusters were divided into different cell types according to marker genes (Fig. S2A, B). Clusters 1, 2, 3, 4, 6, 7, 9, 11, and 13 were annotated as epithelial cells; clusters 0, 5, 8, 10, and 12 were annotated as endothelial cells, T cells, stroma cells, macrophages and B cells (Fig. 1A, B). Trajectory analysis was performed to project all cells from TNBC onto one root and three branches, termed branches 1, 2, and 3 (Fig. 1C, D; Fig. S2C). We found two distinct states of differentiation in epithelial cells, mainly distributed on branches 2 and 3. Simultaneously, in the pseudo-chronological analysis of 14 clusters, we found that some subsets of epithelial cells appeared in almost only one branch, for example, clusters 9 and 13 only appeared in branch 3 (Fig. S2D).

**Figure 1:**
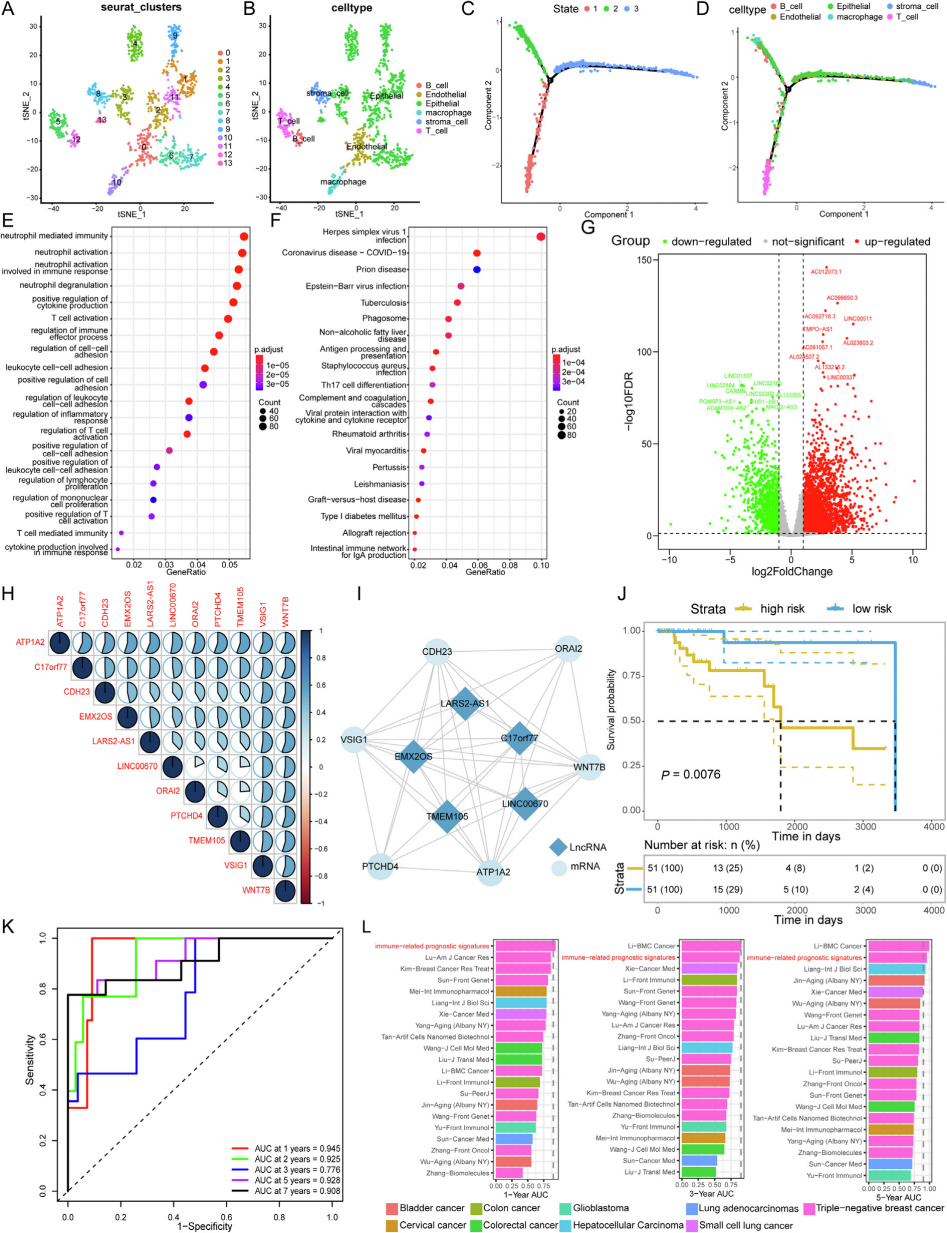
Integrated bulk and single-cell RNA data to construct and validate immune-related prognostic signature models in triple-negative breast cancer. **(A)** Visualization of t-SNE for 14 clusters. **(B)** Visualization of t-SNE for six cell types. **(C)** Distribution of the three states on the branches. **(D)** Distribution of six cell types on branches. **(E)** The dot plots showing the top 20 biological processes of differentially expressed genes (DEGs) between tumor cells and non-tumor cells. The horizontal axis represents the enrichment score of DEGs. The vertical axis represents different GO categories and pathways. The bubble size indicates the number of genes in each category and pathway, and different colors correspond to different log (FDR) values. **(F)** The dot plots showing the top 20 KEGG pathways of differentially expressed genes between tumor cells and non-tumor cells. **(G)** Volcano map of differentially expressed lncRNA. Red and green spots represent significantly upregulated and downregulated lncRNAs. **(H)** Correlation map between the 11 lncRNA-mRNA prognostic signatures. **(I)** Network diagram between the 11 lncRNA-mRNA prognostic signature. **(J)** Kaplan–Meier plots of the OS in the high- and low-risk subgroups of the TCGA cohort. **(K)** The ROC curves of the risk signature in TCGA datasets. **(L)** Comparison of immune-related prognostic signature with other models. The 1-year, 3-year, and 5-year AUC of immune-related prognostic signature and other models developed in the TCGA dataset. The immune-related prognostic signature was highlighted in red.

As mentioned in a previous study, malignant (tumor) cells always accompany a high variable of copy number variation (CNV).^2^ All 1189 cells were used as input for inferCNV, of which B cells, T cells, endothelial cells, macrophages, and stroma cells were used as reference controls (Fig. S3A). We identified a total of 280 tumor cells and 485 non-tumor epithelial cells based on CNV scores. scRNA-seq analysis can improve the study of the genomic heterogeneity of TNBC cells and reveal the specific differentiation status of cancer tumor cells. We visualized tumor cells with other cells and found that tumor cells were mostly present in clusters 1, 2, 4, 7, and 11, while there were almost no tumor cells in clusters 9 and 13 (Fig. S3B). Through another pseudo-chronological analysis, we found that the epithelial cells in state 2 were indeed the tumor cells that we found with inferCNV (Fig. S3C). Therefore, we reasoned that in human epithelial cells, some cells are altered in cancer and become tumor cells. Finally, we used the FindAllmarkers function to perform differential expression analysis on all genes in the cell cluster to identify the differential genes between tumor cells and non-tumor cells. As a result, we identified a total of 129 lncRNAs and 1932 mRNAs (Fig. S3D). These genes were significantly highly expressed in state 2, but were barely expressed in state 1, and showed extremely low expression in state 3 (Fig. S3E). We used GO and KEGG pathway enrichment analysis to show that differential genes were significantly enriched in some immune-related functions and pathways, such as T cell activation, neutrophil-mediated immunity, T cell-mediated immunity, regulation of lymphocyte proliferation, and cytokine production involved in the immune response (Fig. 1E), as well as Th17 cell differentiation and intestinal function immune network for IgA production and other pathways (Fig. 1F). To further explore the immune role of differential lncRNAs and mRNAs, we constructed the co-expression network using co-expression analysis. Then, the genes were assigned to different clusters according to the correlations, resulting in a total of three modules (Fig. S3F). Among the three modules, only 224 hub genes (47 lncRNAs and 177 mRNAs) were identified in the turquoise module.

To identify differentially expressed genes in TNBC compared to normal tissues, we separately analyzed the mRNA expression profiles and lncRNA expression profiles of TNBC from TCGA. We identified a total of 5784 differential mRNAs and 4754 differential lncRNAs, including 3718 up-regulated differential mRNAs and 2066 down-regulated differential mRNAs (Fig. S4A, B), and 3404 up-regulated differential lncRNAs and 1350 down-regulated differential lncRNAs (Fig. 1G; Fig. S4C). We identified 106 lncRNAs and mRNAs that have key roles in the molecular mechanisms of TNBC by integrating bulk and single-cell RNA-seq data to take intersection genes.

Moreover, GO function enrichment analysis demonstrated the enriched biological molecular functions were regulation of activated T cell proliferation, and activated T cell proliferation and excreation (Fig. S4D). In the KEGG pathway enrichment analysis, vascular smooth muscle contraction and gastric acid secretion were the enriched pathways (Fig. S4E). Among these functions and pathways, the key genes in TNBC were found to be significantly enriched in the functions and pathways of T cell immunity, and T cells can recognize tumor cells and initiate an immune response to kill tumor cells. Therefore, these key genes can mediate immune response functions through T cells, thereby inhibiting cancer progression.

To identify potential prognostic signatures in the progression of TNBC, we first applied the LASSO-COX regression model to construct a prognostic model of overall survival in TNBC patients using the gene expression data of 106 key genes in the TCGA dataset. The model identified 11 genes based on the optimal value of λ, obtained five lncRNAs (C17orf77, EMX2OS, LARS2-AS1, LINC00670, TMEM105) and six mRNAs (ATP1A2, CDH23, ORAI2, PTCHD4, VSIG1, WNT7B). We also found that these 11 genes were highly correlated (Fig. 1H, I). LncRNA EMX2OS interacts with mRNA more than other regulatory factors in TNBC patients, which may be a key regulatory factor related to the progression of TNBC ^3^; ORAI2 is up-regulated in TNBC cell lines, and ORAI2 knockdown will block the cell cycle of cancer cells and be more susceptible to cisplatin treatment.^4^ Then we calculate the risk score in the TCGA overall data and the data that randomly divided the TCGA data into the training set and test set at a ratio of 1:1. The samples were divided into high- and low-risk groups according to the median risk score. Kaplan–Meier survival analysis showed that the high-risk cohorts all had worse overall survival than the low-risk cohorts (Fig. 1J; Fig. S4F, G). The ROC curve suggested that the risk score could accurately predict the survival rate of patients (Fig. 1K). Risk scores were highly discriminative for 1-, 2-, 3-, 5-, or 7-year survival, with AUC values of 0.945, 0.925, 0.776, 0.928, and 0.908, respectively. We also performed Kaplan–Meier survival analysis for 11 genes separately and found no significant associations with overall survival time (Fig. S5). Finally, to determine whether the 11 genes could serve as independent prognostic predictors of OS, the risk score was further combined with three clinical and demographic factors (stage, sex, and age) to construct another Cox model. The contributions of risk scores and clinical factors are shown in Figure S4H. Although the stage was significantly associated with overall survival time, the 11 genes were also significantly associated with overall survival time, indicating that the immune-related lncRNA-mRNA prognostic signatures were independent and not affected by other clinical factors.

In addition, we explored the prognostic significance of 11 lncRNA-mRNA signatures in pan-cancer. We obtained expression and clinical data from the TCGA repository for 32 different cancer types, including 8980 patients. The results showed that these 11 lncRNA-mRNA signatures could be used as the prognostic signatures in 15 cancer types (Fig. S6), including adrenocortical carcinoma (ACC, *P* = 3.7e-03), glioblastoma (GBM, *P* = 0.025), head and neck squamous cell carcinoma (HNSC, *P* = 1.2e-03), kidney renal clear cell carcinoma (KIRC, *P* < 1e-04), acute myeloid leukemia (LAML, *P* = 0.045), brain lower grade glioma (LGG, *P* = 0.027), liver hepatocellular carcinoma (LIHC, *P* = 6.2e-04), mesothelioma (MESO, *P* = 0.011), ovarian serous cystadenocarcinoma (OV, *P* = 0.025), pancreatic adenocarcinoma (PAAD, *P* = 7.6e-03), sarcoma (SARC, *P* = 0.024), skin cutaneous melanoma (SKCM, *P* = 1.8e-03), stomach adenocarcinoma (STAD, *P* = 0.049), thymoma (THYM, *P* = 0.041), and uveal melanoma (UVM, *P* = 4.8e-04). We found that the high-risk score groups were associated with a poorer prognosis in 15 TCGA cancer types.

Moreover, to test the prognostic performance of immune-related prognostic signature, we incorporated twenty additional prognostic models for cancer, such as ten prognostic models for TNBC, two prognostic models for bladder cancer, and two prognostic models for colorectal cancer^5^ (Table S1). We found that at 1-year AUC, our immune-related prognostic signature showed better performance than any other prognostic models (AUC = 0.939), and at 3- and 5-years AUC, our immune-related prognostic signature showed better performance than 95% of the other prognostic models (3-years: AUC = 0.880; 5-years: AUC = 0.957; Fig. 1L). Our prognostic prediction model showed compelling clinical value, which will help to improve the overall survival of patients and even help to develop new targeted therapeutic strategies for TNBC patients.

## Author contributions

HYZ, LY and LW designed the study, implemented the algorithm, and performed the analysis. HYZ, LY, LXW, and LW wrote the manuscript. WYL, KLL, XZY and SHL helped to collect the data and prepared the figures and tables. All authors read, reviewed and approved the final manuscript.

## Conflict of interests

The authors declare that the research was conducted in the absence of any commercial or financial relationships that could be construed as a potential conflict of interest.

## Funding

This work was supported by the University Nursing Program for Young Scholars with Creative Talents in Heilongjiang Province, China (No. UNPYSCT-2020174), Excellent Youth Project of Provincial Scientific Research Institute (Heilongjiang, China) (No. CZKYF2022-1-C006), and the Hei Long Jiang Postdoctoral Special Foundation (China) (No. LBH-TZ1018).

## Data availability

All data and codes in this study are available under proper request, please contact wangli@hrbmu.edu.cn.
