# Supplementary material for: Integrated analysis of single-cell and bulk RNA sequencing data reveals immune-related lncRNA-mRNA prognostic signature in triple-negative breast cancer
Source: Genes Dis. 2023 May 10;11(2):571–4. doi: 10.1016/j.gendis.2023.04.006 (PMC10491898; doi:10.1016/j.gendis.2023.04.006)
Supplement: Multimedia component 1 [file mmc1.pdf]

Supplementary Information for

Integrated analysis of single-cell and bulk RNA sequencing data reveals immune-related lncRNA-mRNA prognostic signature in triple-negative breast cancer

Hongying Zhao[1,$], Lei Yu[1,2,$], Lixia Wang [1,$], Xiangzhe Yin[1], Kailai Liu[1], Wangyang Liu[1], Shihua Lin[1], Li Wang[1,*]

***Corresponding Authors:**

Li Wang, College of Bioinformatics Science and Technology, Harbin Medical University, Harbin 150081, China. Tel.: 86-451-86615922, E-mail: wangli@hrbmu.edu.cn

This PDF file includes:

Materials and Methods

Figures S1-S6

Tables S1

Materials and Methods

Data collection

We downloaded the scRNA-seq data of six patients with TNBC (PT039, PT058, PT081, PT084, PT089, and PT126) from the GEO database (GSE118390), which comprised 1,189 high-quality cells with annotated cell types. Additionally, the gene-expression profiles of 113 normal samples and 103 TNBC samples with clinical data were obtained from TCGA (https://cancergenome.nih.gov/) database for differential gene-expression analysis and survival analysis. We also downloaded gene annotation files from the Ensembl (https://asia.ensembl.org/index.html) database for annotating mRNA and lncRNA.

Processing of TNBC scRNA-seq data

A total of 1,189 cells from tumor were included in this analysis. The Seurat package in R was used for quality control, statistical analysis, and exploration of the scRNA-seq data. First, because the GSE118389 dataset had been quality controlled and normalized, we proceeded directly to subsequent analysis. Then, the gene expression of the 1,189 cells was normalized using a linear regression model. Principal component analysis (PCA) was performed to identify significantly available dimensions with a P-value < 0.05. Then, the t-distributed stochastic neighbor embedding (t-SNE) algorithm was applied to conduct dimensionality reduction with nine initial principal components (PCs), as well as to perform cluster classification analysis across all cells. We used the FindClusters function provided by the Seurat package to identify cell clusters and select the resolution. The differential expression

analysis among all genes within cell clusters was performed using the FindMarkers function in Seurat to identify the marker genes of each cluster. An adjusted false discovery rate (FDR) < 0.05 was considered the cutoff criteria for identifying marker genes. Subsequently, different cell clusters were manually identified and annotated according to the cell marker genes.

Trajectory analysis

Pseudotime analysis was performed with Monocle To map differentiation in the TNBC cells and determine the differentiation relationships among cell types and clusters. This algorithm adopts a machine learning technique, learning a parsimonious principal graph to reduce the given high-dimensional expression profiles to a low-dimensional space. Single cells were projected onto this space and ordered into a trajectory with branch points. For data interpretation, the cells in the same branch were generally considered to be in the same differentiation state, while those located in different branches were considered to have different cell differentiation characteristics.

Identification of malignant epithelial cells

Here, we inferred the CNV for the 765 epithelial cells from the gene-expression profiles. The 424 non-epithelial cells were set as a reference set for CNV inference. The genes were sorted by the genomic location, and then the average gene expression of upstream and downstream genes was considered as putative CNV. The inferCNV R package was used for CNV estimation. After inferring the CNV from gene-expression profiles, hierarchy clustering was performed to identify malignant cell clusters. The

clusters with a high variable of CNV were considered malignant cells for subtype identification. The degree of CNV signal in each cell was scored and the cells were plotted on a dendrogram, then cut on the dendrogram, and all epithelial cells were grouped into K clusters (k = 16). We next examined the CNV score for each cluster and found that in clusters 8, 9, 10, 11 and 12 CNV score were relatively low compared to the other clusters and were considered as non-tumor cells.

Differential gene expression analysis

The differentially expressed genes (DEGs) between tumor cells and non-tumor cells were identified from the cell gene expression data using the Seurat R package. The DEGs between TNBC and noncancer tissues were identified from the gene expression data using the edgeR R package. The FDR was applied for multiple testing correction of raw P-values using the FDR method. Fold change (FC) > 2 and FDR < 0.05 were set as the threshold for identifying DEGs.

Identification of module hub genes

The coexpression network for the DEGs was constructed using the WGCNA R package. The network construction procedure included the following main steps: (1) define the similarity matrix; (2) select the weighting coefficient, β, and transform the similarity matrix into an adjacency matrix; (3) transform the adjacency matrix into a topological overlap matrix (TOM); (4) perform hierarchical clustering for TOM-based dissimilarity (dissTOM) to obtain the hierarchical clustering tree; (5) use the dynamic tree cut method to identify the modules from the hierarchical clustering tree and (6) calculate the module eigengene (ME), representing the overall expression level of the

module, of each module. The Pearson correlation coefficients between the MEs of all modules were calculated, and the 1-Pearson correlation coefficient was defined as the average distance between the MEs of all modules. The average-linkage hierarchical clustering method based on a minimum size (gene group) of 30 was employed to cluster the MEs of all modules, and the modules with high similarity were merged to obtain the co-expression network.

Hub genes, which are highly interconnected with the nodes of the module, are of functional importance. Hub gene screening requires the determination of module membership (MM) to measure the correlation between the gene and a given module. For each gene, we defined the MM by the correlation between the gene expression profile and the ME of a given module. For example, $MM^{blue}(i) = cor(x_i, E^{blue})$ measures the correlation between gene i and the ME of the blue module. If $MM^{blue}(i)$ is close to 1 or −1, it is highly connected to the ME of the blue module. However, if $MM^{blue}(i)$ is close to 0, the i-th gene is not part of the blue module. The MM measure is highly related to intramodular connectivity. Highly connected intramodular hub genes tend to have high MM values to the respective module. In short, the larger the MM values of the gene, the higher the correlation between the gene and a given module. In this study, we based on MM (representing the correlation between the gene and a given module) in the WGCNA package to directly identify hub genes. We employed $P < 0.05$ and $MM \geq 0.9$ to obtain hub genes.

Functional enrichment analysis

To obtain the biological functions and signaling pathways of genes, the

clusterProfiler R package was employed for Gene Ontology (GO) annotation and Kyoto Encyclopedia of Genes and Genomes (KEGG) pathway enrichment analysis of the hub genes, respectively. The default parameters were used in the clusterProfiler R package. The thresholds for identification of the GO functions and KEGG pathways of genes were set as FDR < 0.05.

Lasso Regression Model and Risk Survival Analysis

We used LASSO-COX regression algorithm to calculate the risk scores of the key lncRNAs and mRNAs. Finally, the minimum criteria defined the 11 genes and their constants, choosing the perfect penalty parameter λ related to the minimum 10-fold cross validation. We built a multivariate Cox proportional hazards regression model (stepwise method) using the following formula:

$$RiskScore = \sum_{i}^{n} \beta i \times Xi$$

Among them, n represents the number of genes used to establish the multivariate Cox proportional hazards regression model, i represents the digit of the corresponding gene, β is the coefficient of a single gene, and X represents the relative expression of the gene. To identify the prognostic signature by integrating the clinical information of patients suffering from TNBC in TCGA. Kaplan–Meier curve analysis of the samples with key regulatory genes was conducted using the 'survival' package in R. P-values < 0.05 were regarded as significant.

Supplementary Figures

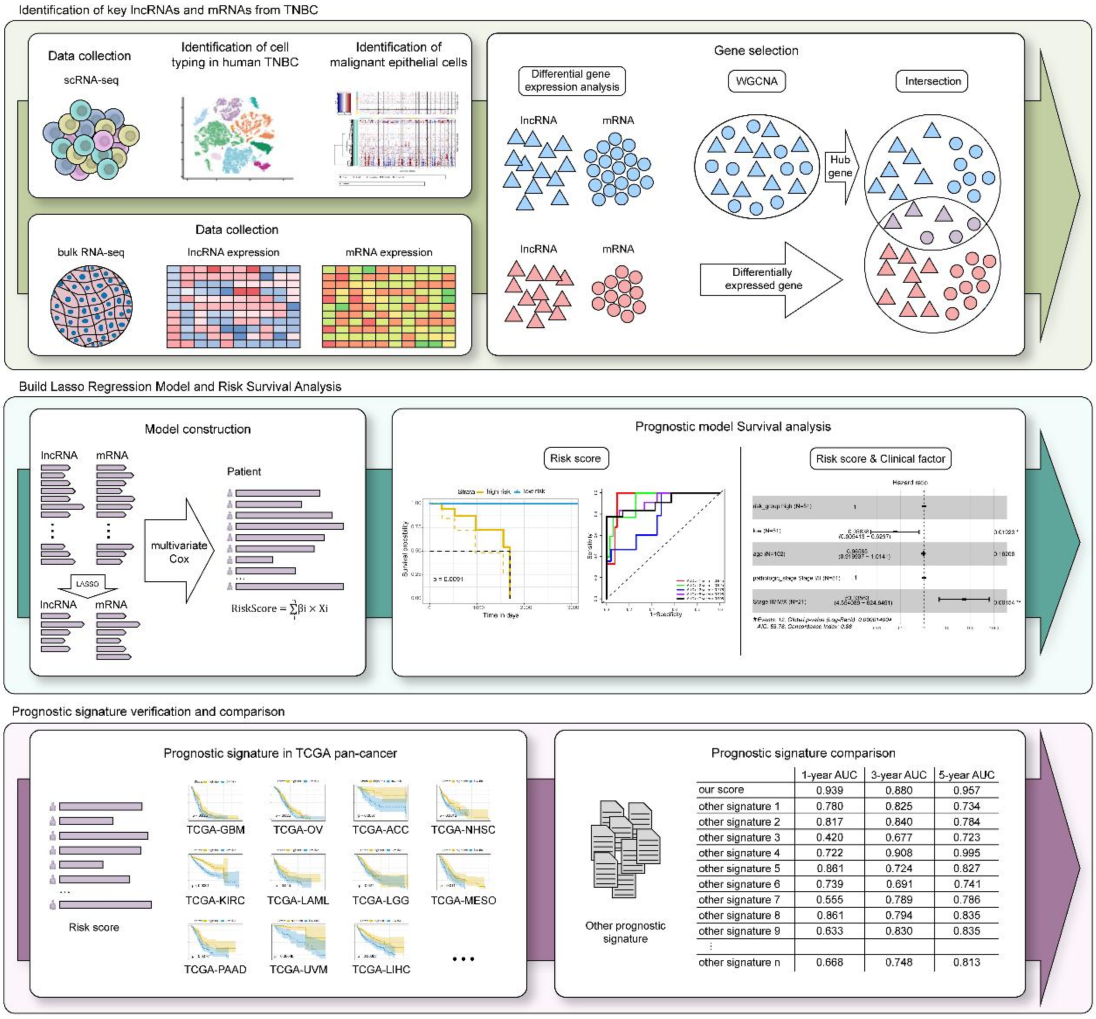

Supplementary Figure 1. Workflow depicting the overall logic of the article.

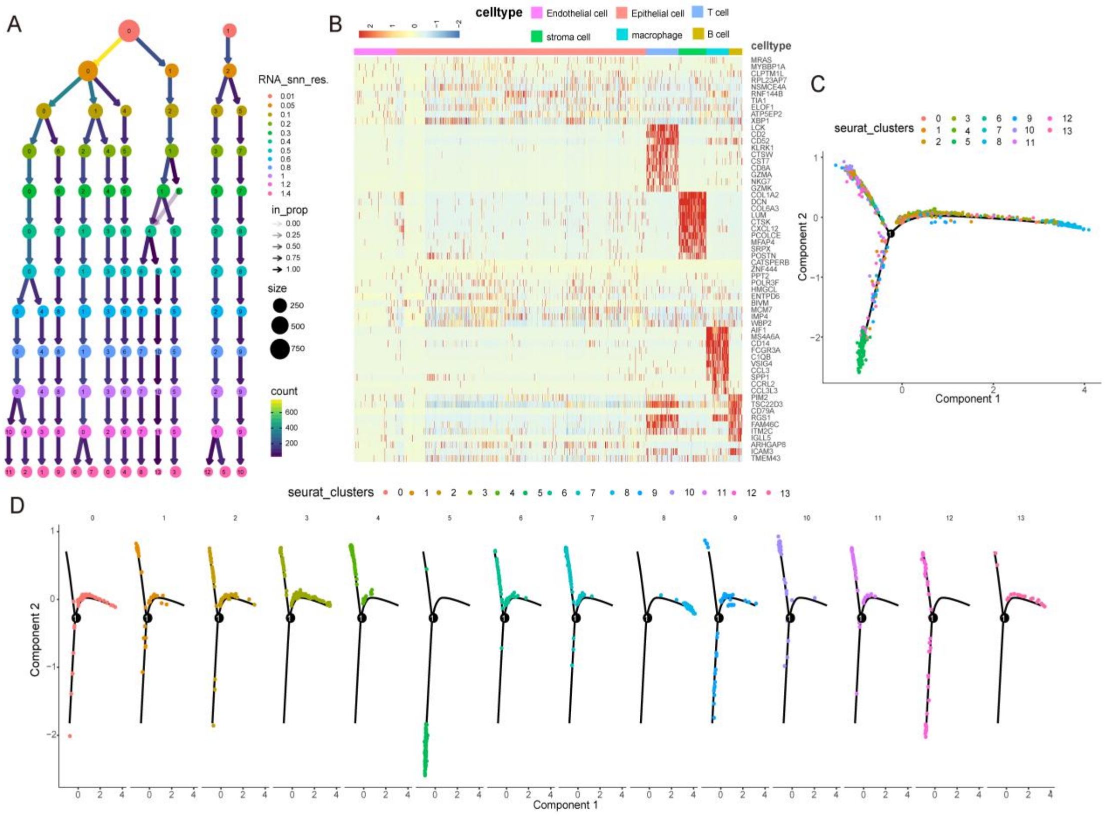

Supplementary Figure 2. Identification of cell types and trajectory differentiation status in TNBC. (A) The dendrogram shows that the clusters are divided by choosing an appropriate resolution. (B) The top 10 marker genes of each cell type are displayed in the heatmap. (C) Distribution of 14 clusters on branches. (F)Distribution of the three states on the branches. (D) The distribution of each of the 14 clusters on the branches.

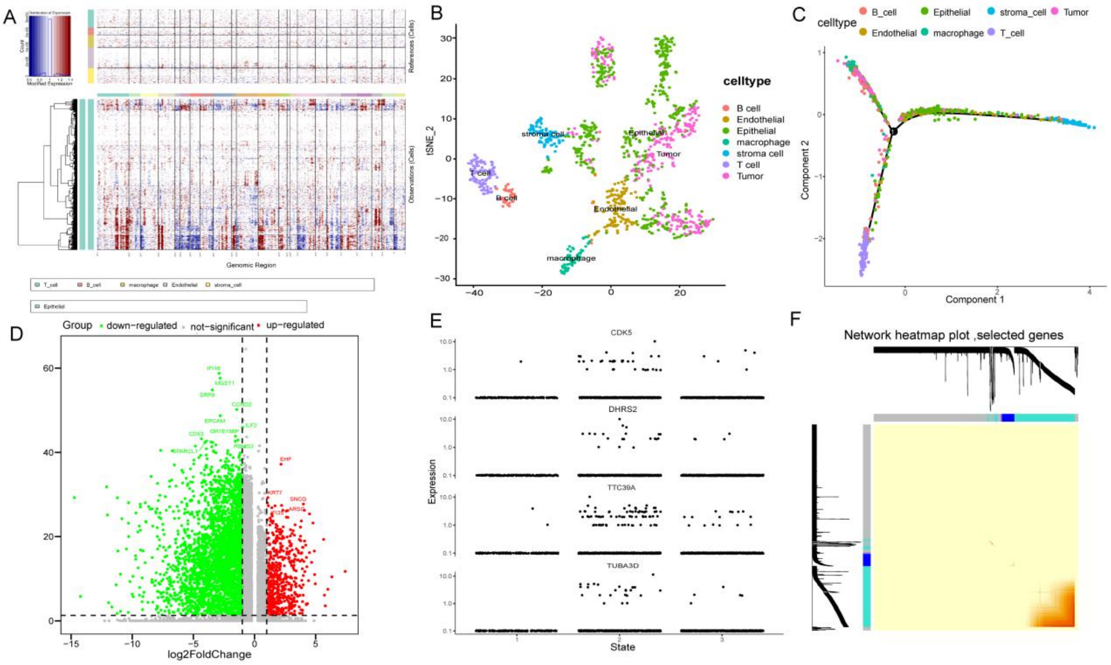

Supplementary Figure 3. Identification of TNBC tumor cells and differentiation of specific states. (A) Heatmap of the inferred CNV across 765 epithelial cells, in which genes were sorted by genomic location. (B) Visualization of the seven cell types based on t-SNE. (C) Trajectory analysis for the seven cell types. (D) Volcano map of differentially expressed gene. Red and green spots represent significant upregulated and downregulated mRNAs. (E) Gene expression levels of CDK5, DHRS2, TTC39A, and TUBA3D genes in states 1, 2 and 3. (F) Heatmap plot for visualizing the gene network. The heatmap depicts the TOM among all DEGs in the analysis. Darker red indicates a greater overlap, while the light color indicates a low overlap; the modules are represented by the blocks of darker color along the diagonal. The module assignment gene and dendrogram are shown along the top and left sides.

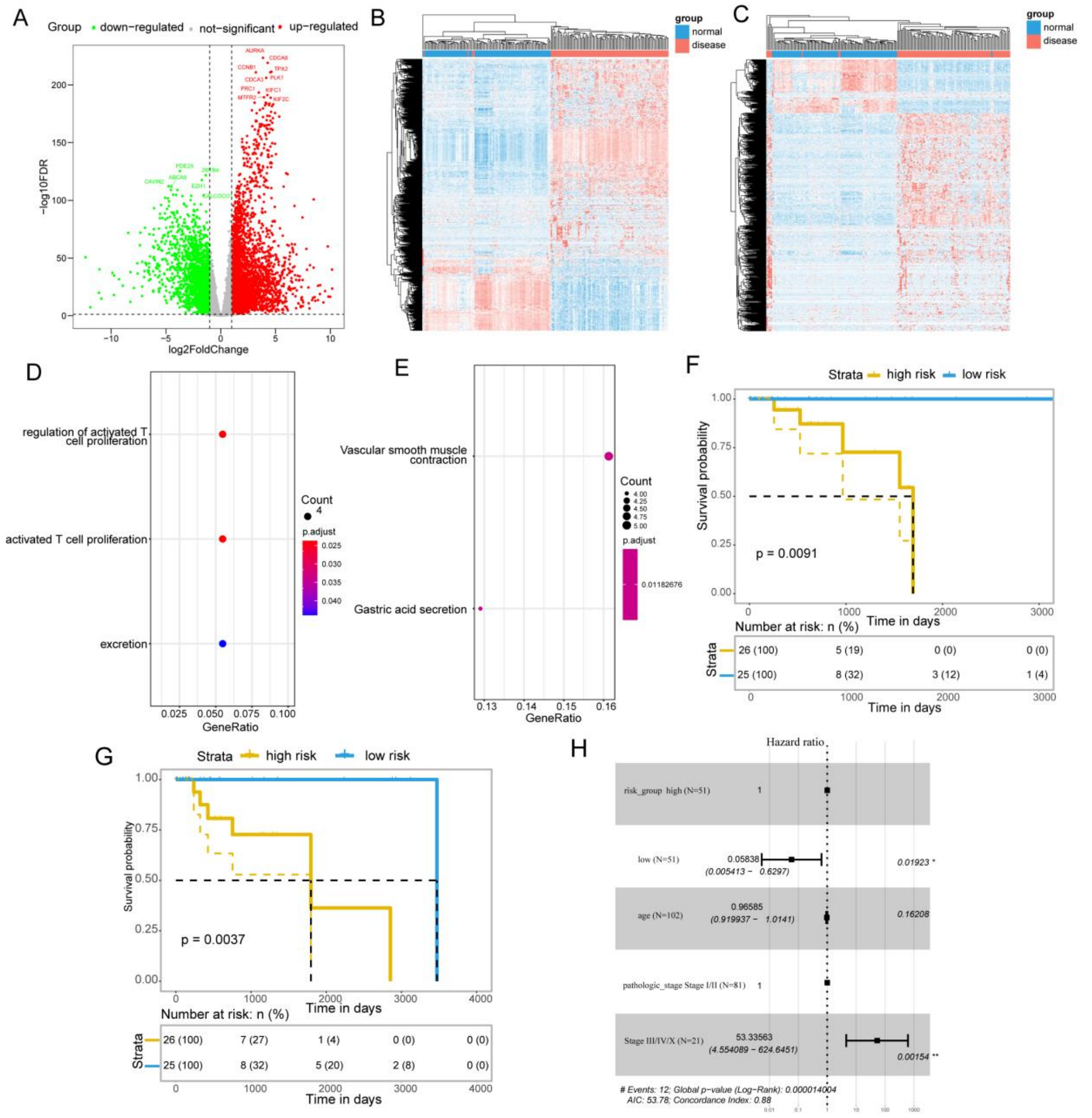

Supplementary Figure 4. **Identification of key lncrna and mrna in batch RNA-seq data and construction of prognostic models.** (A) Volcano map of differentially expressed mRNA. Red and green spots represent significantly upregulated and downregulated RNAs, respectively. (B-C) Heatmap of differentially expressed mRNAs (B) and lncRNAs (C). (D) Dotplot showing the top three biological processes of 106 key genes. (E) Dotplot showing the top two KEGG pathways of 106 key genes. (F) Kaplan–Meier plots of the OS in the high- and low-risk subgroups of TCGA training set cohort. (G) Kaplan–Meier plots of the OS in the high- and low-risk subgroups of TCGA test set cohort. (H) Multivariate Cox regression analyses of the association between clinicopathological factors (including the risk score) and overall survival of patients in TCGA datasets.

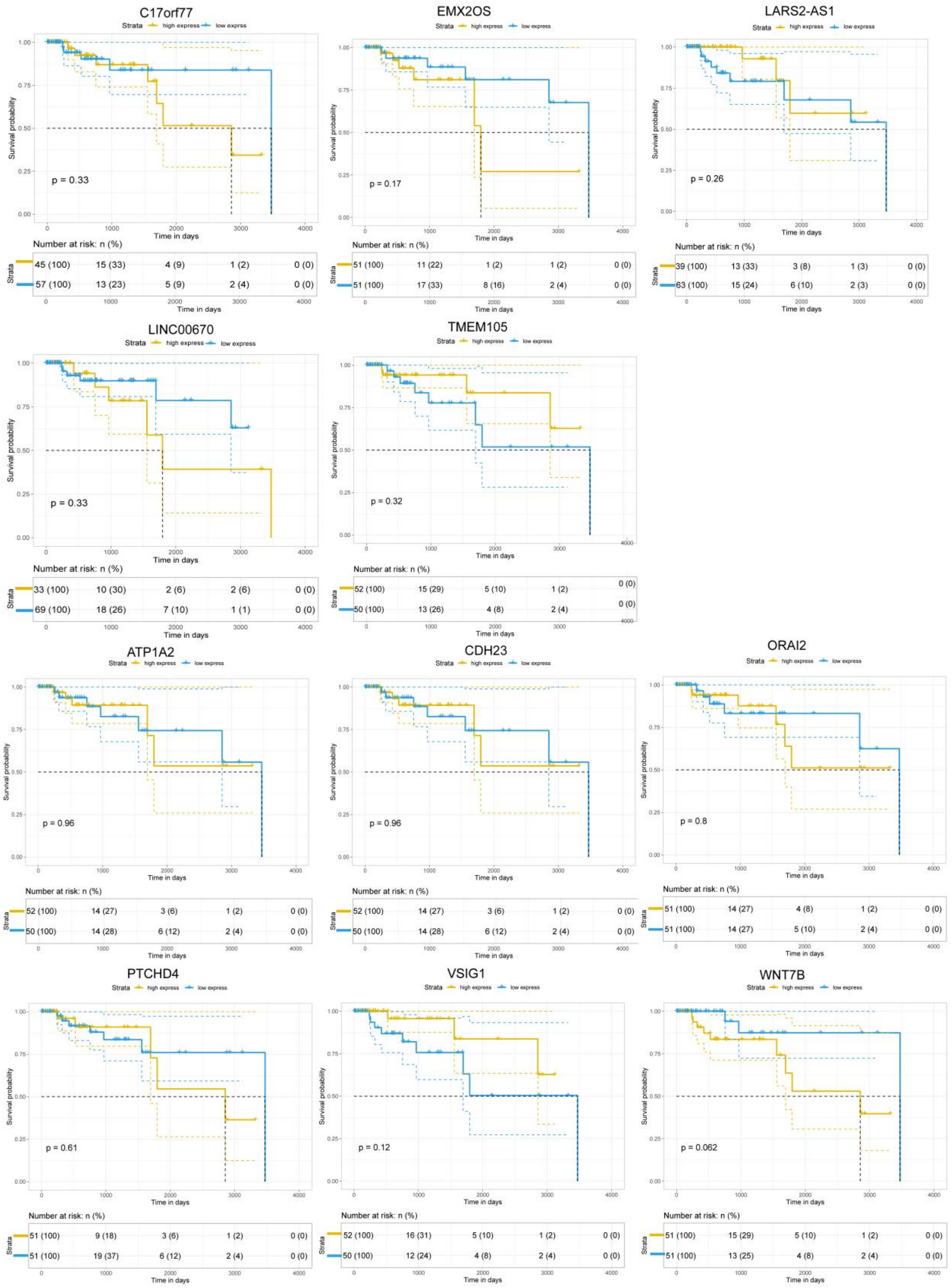

Supplementary Figure 5. **Kaplan-meier plots of five lncRNAs and six mRNAs as independent prognostic factors.**

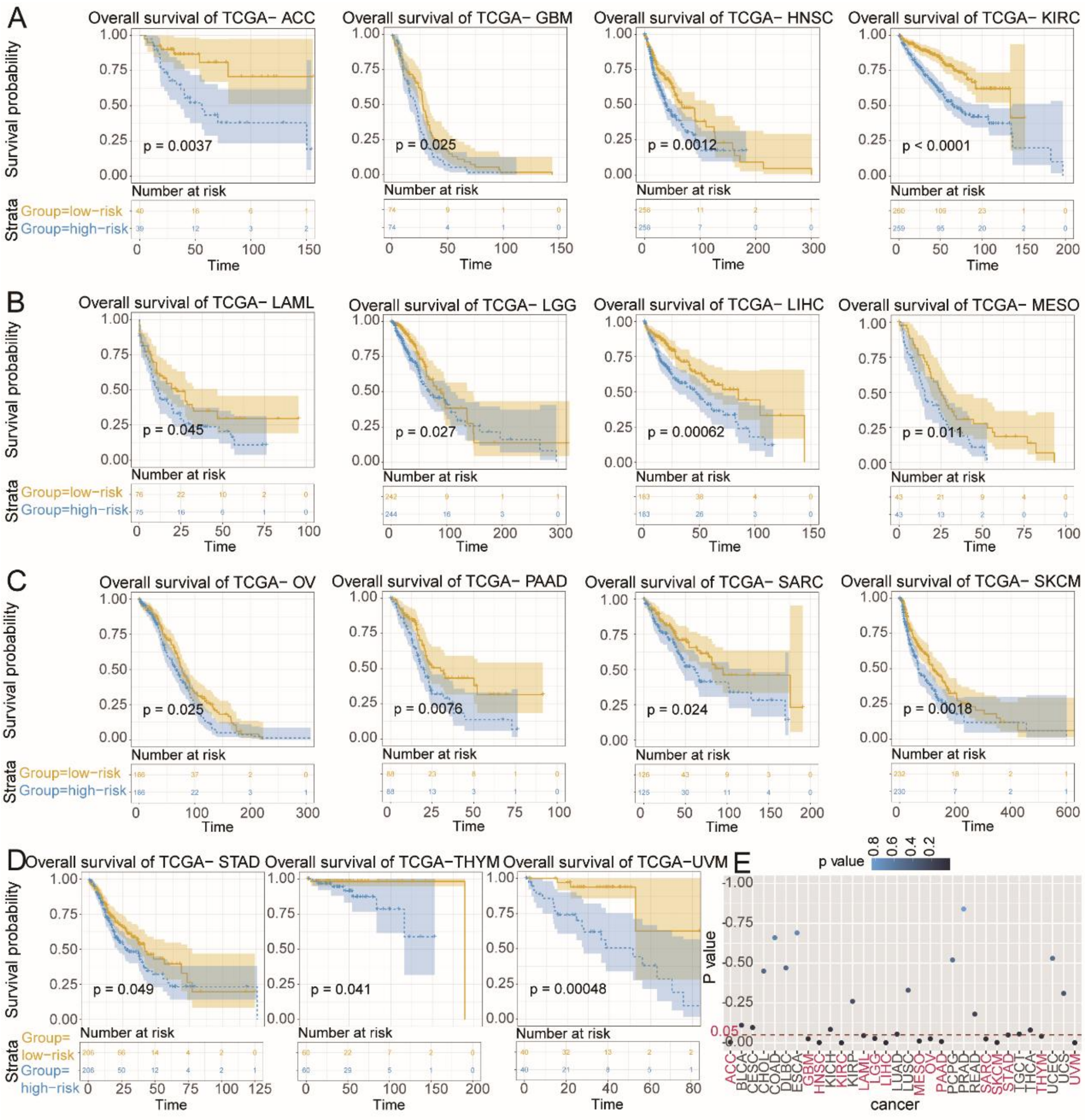

Supplementary Figure 6. **Survival analysis of 11 lncRNA-mRNA in pan-cancer.** Kaplan-Meier plot curves and risk score of overall survival based on risk scores of the 11 genes in 15 cancer types including ACC, GBM (A), HNSC, KIRC, LAML, LGG (B), LIHC, MESO, OV, PAAD (C), SARC, SKCM, STAD, THYM and UVM (D) from TCGA. The P-values was calculated using the log-rank test. (E) A total of 32 types of cancer were analyzed. The red dotted line represents a P-value equal to 0.05. Filled colors from dark blue to light blue represent P-values.

Supplementary Table 1: 20 additional prognostic models for cancer

Cancer	Prognostic model	Reference
Triple-negative breast cancer	SERPINE1, IL2RG, CXCL11, CXCL13, LRSAM1, TAPBPL	Yang, Xia et al. (2021)
Triple-negative breast cancer	HSPA6, LCN1, ARTN, IL36G, BCL2A1, CASP12	Sun, Xiaorui, and Tiansong Zhang. (2022)
Triple-negative breast cancer	TAPBPL, FBP1, GPRC5C	Zhang, Jinguo et al.(2022)
Triple-negative breast cancer	EGR3, CCND2, SOCS3, JUND, SLC27A6	Li, Xiao-Fen et al. (2022)
Triple-negative breast cancer	CD1B, CD53, CT45A1, GTF3C1, IL11RA, IL1RN, LRRN3, MAPK1, NEFL, PRKCE, PTPRC, SPACA3, TNFSF11	Kim, Ji-Yeon et al.(2020)
Triple-negative breast cancer	IGHG2, IGHJ2, IGKV1D-39, NRAS, PIK3CA	Tan, Weige et al. (2020)
Triple-negative breast cancer	BIRC3, BTN3A1, CSF2RB, GIMAP7, GZMB, HCLS1 ,LCP2, SELL	Zhang, Jinguo et al. (2020)
Triple-negative breast cancer	CA9, C7orf68, PARM1, MATK, PLCL2, HLA-DRB4, RAMP3, TMEM176A, COBL, STAMBPL1, KLRD1, PHF15	Lu, Xunxi et al. (2022)
Triple-negative breast cancer	RMND5A, ZNF829, KDM5B, NCBP2, GPI, BGN, BGN, CCND2, PLBD1, ZYG11A, IL17RD	Wang, Xiangru, and Hanghang Chen.(2022)
Triple-negative breast cancer	CD79A, CXCL13, IGLL5, LHFPL2, PLEKHF1	Su, Peng et al. (2021)
Hepatocellular Carcinoma	SLC7A11, G6PD, CISD1, CARS, SLC1A5, ACACA, ACSL3, NQO1, NFS1, GPX4	Liang,Jie-Ying et al.(2020)
Colorectal cancer	SLC10A2, FGF2, CCL28, NDRG1, ESM1, UCN, UTS2, TRDC	Wang, Jun et al. (2020)
Bladder cancer	MMP9, RBP7, ADIPOQ, AHNAK, OAS1, RAC3, SLIT2, EDNRA, IL34, PDGFD, PPY, IL17RD	Jin, Kun et al. (2021)
Lung adenocarcinomas	ARNTL2, ECT2, PPIA, TUBA4A	Sun, Sijin et al. (2020)
Glioblastoma	H19, AL162231.2, AC002456.1, ST3GAL6-AS1, SOX21-AS1, AC006213.5	Yu, Wanli et al. (2021)
Colorectal cancer	VGF, RLN3, CT45A1	Liu, Jungang et al.(2021)
Cervical cancer	RIPOR2, DAAM2, SORBS1, CXCL8	Mei, Jie et al. (2020)

Bladder cancer	MIR181A2HG, AC114730.3, LINC00892, PTPRD-AS1, LINC01013, MRPL23-AS1, LINC01395, AC002454.1	Wu, Yucai et al. (2020)
Colon cancer	CEBPB, CXCL9, IRF8, ITGB1, LAG3, MCFD2, PSMD11, RNASE7, SPARC, TAP2	Li, Xinyu et al. (2020)
Small cell lung cancer	NR3C1, NR1D2, TANK, ARAF, HDGF, INHBE, LRSAM1, PLXNA1, PML, SP1	Xie, Qi et al. (2021)
